# Interspecies and temporal dynamics of bacterial and fungal microbiomes of pistil stigmas in flowers in holoparasitic plants of the *Orobanche* series *Alsaticae* (Orobanchaceae)

**DOI:** 10.1038/s41598-023-33676-0

**Published:** 2023-04-25

**Authors:** Karolina Ruraż, Sebastian Wojciech Przemieniecki, Renata Piwowarczyk

**Affiliations:** 1 Center for Research and Conservation of Biodiversity, Department of Environmental Biology, Institute of Biology, Jan Kochanowski University, Uniwersytecka 7, 25-406 Kielce, Poland; 2 Department of Entomology, Phytopathology and Molecular Diagnostics, University of Warmia and Mazury in Olsztyn, Prawocheńskiego 17, 10-720 Olsztyn, Poland

**Keywords:** Ecology, Microbiology, Plant sciences

## Abstract

Little is known about the microbiomes of flower parts, and even less information is available regarding these microorganisms’ colonization of specific niches in parasitic plants. We investigate the temporal interspecies dynamics of the parasitic plants microbiome of flower stigmas in two stages of development: immature stigmas in flower buds and mature stigmas in opened flowers. We compared two related holoparasitic *Orobanche* species from localities approximately 90 km apart and characterize their bacterial and fungal communities using 16S rRNA gene and ITS sequences, respectively. We identified from 127 to over 228 OTUs per sample for fungi, sequences belonging to genera: *Aureobasidium*, *Cladosporium*, *Malassezia*, *Mycosphaerella*, and Pleosporales, constituting approximately 53% of the community in total. In the bacterial profile, we recorded 40 to over 68 OTUs per sample consisting of Enterobacteriaceae, and genera *Cellulosimicrobium*, *Pantoea*, and *Pseudomonas* spp., with an approximately 75% frequency. In microbial communities, higher numbers of OTUs colonizing mature stigmas were recorded than in immature. This implies that the dynamics and concurrence of microbial communities were different between *O. alsatica* and *O. bartlingii* and underwent significant changes during flower development. To the best of our knowledge, is the first study of the interspecies and temporal dynamics of the bacterial and fungal microbiomes of pistil stigmas in flowers.

## Introduction

Orobanchaceae is the largest parasitic family and includes over 102 genera and 2100 species^1^. These species include mainly semi-parasitic and holoparasitic plants, which extract nutrients from the roots of the host plants by the presence of special structures called haustoria. Some are dangerous weeds of economic importance. Many holoparasitic species are rare and endangered plants in countries in Europe and Asia^2–4^. The tribe Orobancheae, the oldest and most species-rich lineage of holoparasitic Orobanchaceae, has a worldwide distribution but is concentrated in the warmer parts of Europe, especially in the Mediterranean Basin, western and central Asia, and northern Africa^2,5^. In this study, we used *Orobanche alsatica* Kirschl. and *O. bartlingii* Griseb. which are Eurasian species. The range of *O. alsatica* extends from eastern France through central Europe to China, with numerous localities in central Europe. Similarly, the range of *O. bartlingii* is from western, central and eastern Europe to China, with a main range in the Baltic states, Russia to Siberia, and the Caucasus^3,6^. *O. alsatica* is an endangered species in Poland and critically endangered in Germany, endangered in the Czech Republic and vulnerable in Slovakia. *O. bartlingii* is vulnerable in Poland, endangered in Germany, the Czech Republic and Slovakia, and is included in *O. alsatica* aggr. and has the same status. In Poland, these species occur only in a few localities, mostly in S and SE Poland (Silesian-Kraków Upland, Małopolska Upland, Lublin Upland, Central Roztocze and Małe Polesie region)^7,8^. Holoparasitic plants lack chlorophyll (and are thus nonphotosynthetic), most often producing impressive, colourful inflorescences, and their life strategy is strongly focused on achieving the greatest reproductive success through pollination and thus the production of many seeds called ‘dust seeds’^4,9^. These species become visible above the surface of the soil only at the time of flowering. Most Orobanchaceae species have a single inflorescence, and the maturation of reproductive structures starts in the bottom region followed by the middle and top regions of the inflorescence. Broomrapes usually have a short flowering time, during which we can observe closed flower buds as well as fully developed flowers with a prominent stigma on one individual^6^. Flowers of parasitic plants are bisexual and show a number of adaptations to insect pollination, e.g., small flowers gathered in dense inflorescences with a contrasting colouration and shine of the corolla and stigma^10–13^.

The stigma is a part of the pistil connected to the ovary, receiving pollen during pollination. It is a transitional structure, and its development usually takes several days. Depending on the structure of the pistil and the pollination biology of a particular species, it may have different shapes, structures and receptive epidermal products that facilitate the retention of pollen grains on the stigmas^14^. Thus, it is a crucial structure in the plant reproductive process. The surface of the stigmas is a very nutrient-rich environment containing sugars, amino acids and phenolic and lipid compounds^15,16^. Therefore, the stigma is in contact with diverse microorganisms originating either through soil, air, water or insects. Moreover, it can influence its microbiome by secreting various metabolites, and the microbiome may also control the metabolome of the host plant^17^. Increased insight into the composition and function of plant microbiota requires investigation of diverse ecological niches.

Microorganisms, including bacteria, fungi, protists, nematodes and viruses, can play a significant role in plant health by facilitating growth promotion, nutrient uptake, stress tolerance and resistance to pathogens^18,19^. However, most studies have mainly investigated the composition of the phyllosphere and rhizosphere microbiota^20–22^. In contrast, analyses of microbiota associated with the anthosphere (an adjacent zone around the flowers) have been relatively less observed. Endophytic microorganisms are protected from the outside environment on the one hand but are easily affected by changes occurring in the plant tissue on the other. This is in contrast to epiphytic microbial colonization of the stigmas, which cannot be protected from dehydration and pathogen attack in the form of thick cuticle or wax because they must capture and hydrate pollen. These microhabitats have their own and specific compositions of associated microorganisms, and some florivores and insect-like pollinators may dynamically change this niche^23^. The parts of flowers are a unique environment for microbial communities because they contain multiple niches differing in morphology, chemical composition and longevity. The genera *Metschnikowia* (Ascomycota), *Cryptococcus* (Basidiomycota), and *Pseudomonas* and *Acinetobacter* (Proteobacteria) are very commonly detected as a members of the flower microbiome across a variety of plants. The best known among the microbial community are yeasts inhabiting nectar^24^. The floral microbiome is unique and different from the microbiome of other plant parts, a few bacterial taxa that were not detected in other parts of the plant^25^. In addition, research across different parts of flowers indicates separation in the microbial community structure within the flower^26^. Previous studies have shown a small number of reports on the stigma microbiome, mainly focusing on apple stigmas^27,28^. Although certain lineages are predominantly within the phylum Proteobacteria, they tend to be dominant, especially with the majority of sequences belonging to Enterobacteriaceae and Pseudomonadaceae. The apple flower microbiome also contained many taxa affiliated with Actinobacteria, and Bacteroidetes^29–31^.

The composition and role of bacteria and fungi colonization of the flower parts of species from tribe Orobancheae is relatively poorly understood. Bacteria identified in the pre-haustorium stage, tubercle (spider stage) and shoot of *Phelipanche aegyptiaca* (Pers.) Pomel generally have been recognized as from among the Proteobacteria, Actinobacteria, Bacilli, Flavobacteria, and Sphingobacteria, but bacterial communities change at different stages of parasitism^32^. Research conducted on *Orobanche hederae* Vaucher ex Duby has shown that the parasitic plant microbiome is derived but distinct from the host plant microbiota. Moreover, the results of a recent study between *Cistanche deserticola* Ma and hosts *Haloxylon ammodendron* (C.A.Mey.) Bunge ex Fenzl suggested that the root microbiota of the parasitic plant was highly congruent with those of the host plant^33^. The bacterial profile of parasitic roots (haustoria) mainly consists of Proteobacteria, Bacteroidetes, Acidobacteria, Actinobacteria, Nitrospirae and Verrucomicrobia, similar to leaves (scales), in which the presence of Firmicutes, Planctomycetes and Chloroflexi has been noted^34^. Therefore, it seems that rich bacterial communities play an equally important role in the broomrape life cycle. *Phelipanche ramosa* (L.) Pomel seed core microbiota are mainly represented by four phyla of bacteria: Proteobacteria, Bacteroidetes, Actinobacteria and Firmicutes. Additionally, six fungi genera were present representing 64.43% of the total abundance: *Cladosporium*, *Fusarium*, *Gliocladium*, *Mycosphaerella*, *Plectosphaerella*, and *Vishniacozyma*^35^. The endophytic bacteria from *Phelipanche ramosa* seeds are closely related to *Brevibacterium frigoritolerans* and *Bacillus simplex*, described as soil bacteria that are highly resistant to environmental conditions, and the plant growth-promoting rhizobacteria have been identified^36^. For the *Cistanche armena* (K. Koch) M. V. Agab. seeds, confirmed Proteobacteria, Firmicutes and Actinobacteriota, followed by Bacteroidetes, Acidobacteria, Verrucomicrobia, Mixococcota, Planctomycetes, Patescibacteria and Chloroflexi were less abundant^37^. In floral nectar, bacteria and yeasts such as Enterobacteriaceae in *Orobanche rapum-genistae* Thuill., and *Pseudomonas* sp. and *Cryptococcus* sp. have been observed in *Phelipanche ramosa*^38^.

Despite these reports, to date there is no published information regarding the bacteria and fungi inhabiting the stigmas of holoparasitic plants from the Orobanchaceae. These studies, never before carried out on parasitic plants, provide an opportunity to learn about the processes influencing their reproductive success, including variability in the microbiological profiles of pistil stigmas depending on their stages of development and taxonomic affiliations.

The aim of this study is to determine the composition and diversity of the microorganisms (bacteria and fungi) of the flower stigma in related complex holoparasitic plants of the *Orobanche* series *Alsaticae* (Orobanchaceae), concerning the central European representatives of the *O. alsatica* aggregate, parasitizing Apiaceae species, including *O. alsatica* and *O. bartlingii*. We investigate the microbiological profile of the stigma by distinguishing differences and similarities, i.e., at the interspecies level by using molecular methods—Next Generation Sequencing (NGS). Additionally, we examine and compare how dynamics of microorganisms were shaped between the two species separated around 90 km (the interspecies dynamics), as well as in the development of stigma in the case of immature stigmas from closed flower buds without access to the external environment and from mature stigmas from opened flowers that had access to the external environment (the temporal dynamics).

## Results

After quality and length filtering, 297,043 bacterial 16S rRNA reads (from 26,105 to 48,759 per sample) and 506,384 fungal ITS reads (from 46,827 to 81,669 per sample) were obtained from the samples. The number of unfiltered reads that were aligned to these OTUs ranged from 40 to over 68 per sample for bacteria and from 127 to over 228 per sample for fungi. In both microbial communities, higher numbers of OTUs colonizing mature stigmas were recorded compared with immature stigmas in *Orobanche alsatica* and *O. bartlingii* (Table 1).

**Table 1:** OTUs and diversity indices of microbiota identified in *Orobanche alsatica* (OA) and *O. bartlingii* (OB) in immature stigmas from closed flowers (1, 2, 5, 6) (IS) and mature stigmas from opened flowers (3, 4, 7, 8) (MS) and statistical calculation.

Plant species	*O. alsatica*				*O. bartlingii*				Significances (p-value)	
Stigma maturity	Immature stigmas		Mature stigmas		Immature stigmas		Mature stigmas			
Measure	OA1	OA2	OA3	OA4	OB5	OB6	OB7	OB8	OA × OB*	IS × MS*
Bacteria										
OTUs	51	40	62	48	51	65	56	68	0.156	0.352
Simpson’s dominance (λ)	0.31	0.47	0.25	0.29	0.65	0.61	0.19	0.17	0.609	**0.028**
S–W diversity (H′)	1.54	1.20	1.78	1.69	0.89	0.98	2.10	2.19	0.974	**0.006**
Pielou’s evenness (J′)	0.39	0.33	0.43	0.44	0.23	0.24	0.52	0.52	0.824	**0.010**
Fungi										
OTUs	131	127	228	196	142	188	161	212	0.863	**0.040**
Simpson’s dominance (λ)	0.09	0.11	0.07	0.07	0.08	0.09	0.12	0.22	0.257	0.971
S–W diversity (H′ )	2.97	2.88	3.30	3.23	3.03	3.13	2.85	2.22	0.254	0.710
Pielou’s evenness (J′ )	0.61	0.60	0.61	0.61	0.61	0.60	0.56	0.42	0.257	0.657

*Only OTUs with significant biological differences (*P* < 0.05) were marked. Significant values are in [bold].

### Bacterial and fungal abundance and dominance

**Figure 1:**
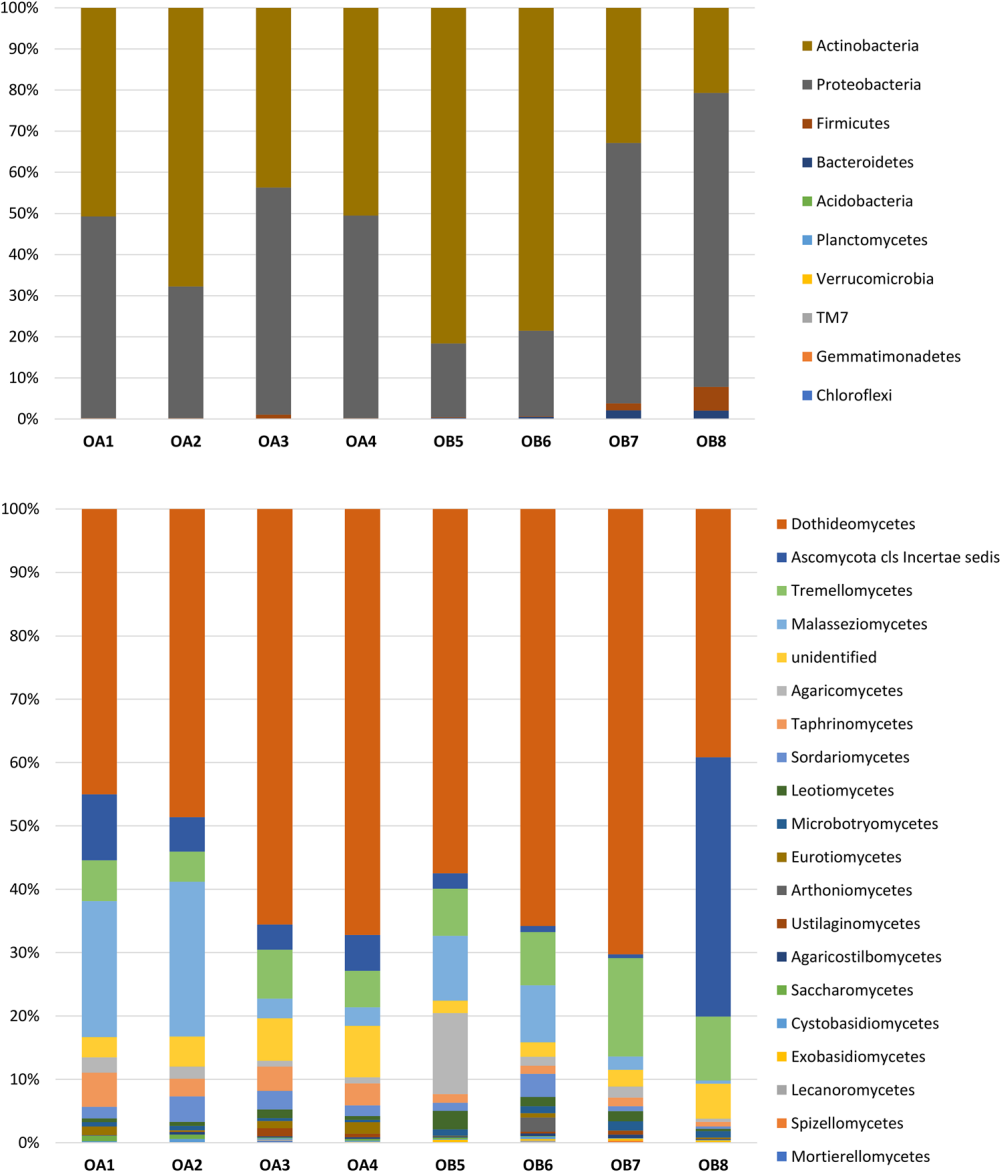
Changes in the composition of bacterial (phylum level; top) and fungal (class level; bottom) communities inhabiting *Orobanche alsatica* (OA) and *O. bartlingii* (OB) in immature stigmas from closed flowers (1, 2, 5, 6) and mature stigmas from opened flowers (3, 4, 7, 8).

The microbiome was composed mainly of the phyla Actinobacteria and Proteobacteria, which constituted approximately 98% of the community (Fig. 1). The analysis of the examined stigma samples allowed us to identify bacteria representing the 15 most numerous OTUs (Table 2), including *Cellulosimicrobium* sp., *Pantoea ananatis*, Enterobacteriaceae, *Pseudomonas* spp., *Stenotrophomonas* spp., Rickettsiales, and *Pseudomonas fragi*, constituting approximately 93.27% of the community in total. The studies demonstrated eudominance or dominance of *Cellulosimicrobium* sp. (19.62–79.61%). The dominant bacteria included *Pantoea ananatis* (16.50 and 22.83%) as well as Enterobacteriaceae (22.57 and 30.86%), *Pseudomonas* spp. (10.08, 11.83 and 12.27%), *Stenotrophomonas* spp. (10.33 and 12.35%), Rickettsiales (17.59%), and *Pseudomonas fragi* (13.68 and 15.84%).

**Table 2:**
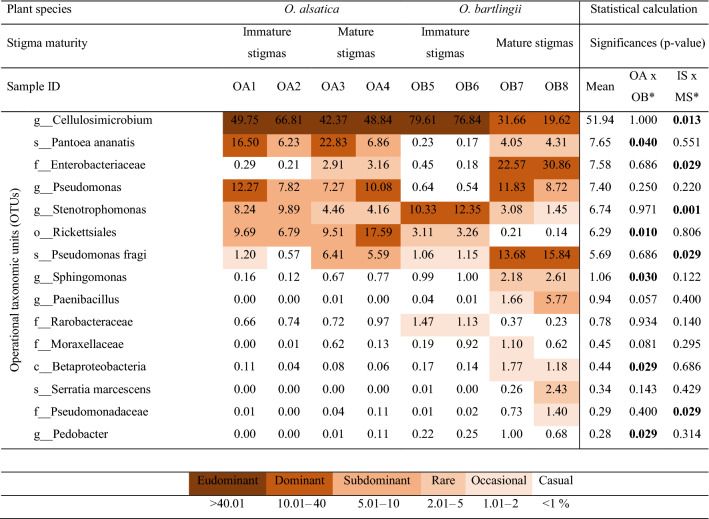
Frequency (%) of bacteria identified in *Orobanche alsatica* (OA) and *O. bartlingii* (OB) in immature stigmas from closed flowers (1, 2, 5, 6) (IS) and mature stigmas from opened flowers (3, 4, 7, 8) (MS) and statistical calculation.

*Only OTUs with significant biological differences (*P* < 0.05) were marked. Significant values are in [bold].

The dominant class of fungi (approximately 57%) in the analysed stigmas was Dothideomycetes, followed by Ascomycota cls Incertae sedis (9.8%), Tremellomycetes (8.6%) and Malasseziomycetes (8.3%) (Fig. 1). Analysis of the composition of fungi present on the stigmas showed the presence of 36 of the most numerous OTUs (Table 3). We noted high abundance—70.45% of the community in total of *Mycosphaerella tassiana*, *Cladosporium delicatulum*, *Aureobasidium pullulans*, *Malassezia restricta*, Pleosporales, *Tetracladium* spp., *Chalastospora ellipsoidea*, *Vishniacozyma victoriae* and *Volucrispora graminea*. The eudominant and dominant were represented by *Mycosphaerella tassiana* (11.01–25.51%), *Cladosporium delicatulum* (10.39–19.49%), *Aureobasidium pullulans* (12.11–16.63%), *Malassezia restricta* (17.47 and 22.88%), Pleosporales (12.49%), *Tetracladium* spp. (40.82%), *Volucrispora graminea* (10.4%), and Ceratobasidiaceae (10.34%).

**Table 3:**
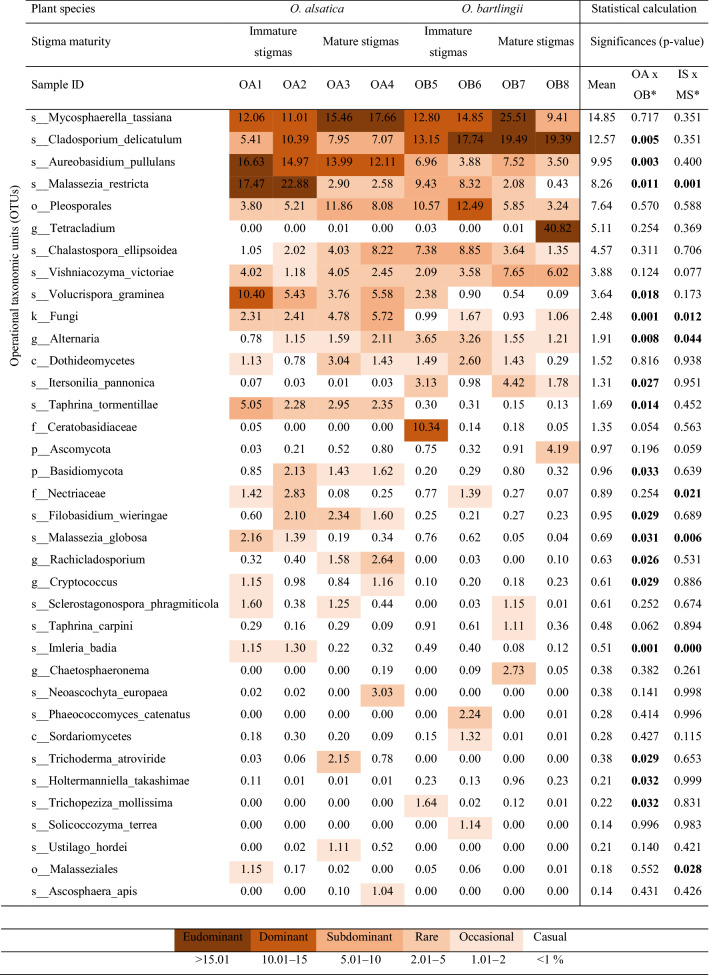
Frequency (%) of fungi identified in *Orobanche alsatica* (OA) and *O. bartlingii* (OB) in immature stigmas from closed flowers (1, 2, 5, 6) (IS) and mature stigmas from opened flowers (3, 4, 7, 8) (MS) and statistical calculation.

*Only OTUs with significant biological differences (*P* < 0.05) were marked. Significant values are in [bold].

### Ecological indices

Analysis of ecological indicators of bacterial communities in stigmas of pistil at different stages of development revealed similar relationships, as did the analysis of fungal communities. In the fungal community of the stigma, the values of Simpson’s dominance, Shannon diversity and Pielou’s evenness indices for the communities were more similar than those for bacteria. Statistical analysis showed significant differences between bacteria in immature and mature stigmas in the case of the analysed indicators, as well as between fungi significant differences were recorded only for OTUs (Table 1). A lower value for the Simpson index in bacteria was found in the case of mature stigmas in both *Orobanche alsatica* (average λ = 0.27 vs. 0.39) and *O. bartlingii* (λ = 0.18 vs. 0.63), which demonstrates a greater species diversity. In the case of fungi, the situation was similar in *O. alsatica* (λ = 0.07 vs. 0.10), while in *O. bartlingii* (λ = 0.17 vs. 0.08) in mature stigma, the index was slightly higher. In the case of the Shannon diversity index, there was an inverse relationship. The index values were higher in the case of bacterial communities comparing immature to mature stigmas (H′ = 1.37 vs. 1.73 for *O. alsatica* and H′ = 0.94 vs. 2.14 for *O. bartlingii*) in both species. The situation was similar in the case of this index in relation to the Simpson index, i.e., for *O. alsatica* H′ = 2.92 vs. and 3.26 and for *O. bartlingii* H′ = 3.08 vs. and 2.54. The Pielou’s J evenness index in bacteria was 0.36 vs. 0.43 for *O. alsatica* and 0.23 vs. 0.52 for *O. bartlingii* and in immature and mature stigmas, respectively. However, in fungal communities, this index achieved a similar value (approximately 0.60) for *O. alsatica* in both stages and for *O. bartlingii* in immature stigmas, but in mature stigmas, *O. bartlingii* had a lower value (0.49) (Table 1).

### Interspecies diversity in the *Orobanche alsatica* and *O. bartlingii* microbiomes at different stages of flower development

Taking into account the differentiation of bacterial microorganisms colonizing the stigmas of analysed species, it should be noted that the incidence of the most numerous bacteria was almost identical, i.e., *Cellulosimicrobium* sp. for *Orobanche alsatica* (51.94%) versus *O. bartlingii* (51.93%). A similar distribution was presented for *Stenotrophomonas* spp. (6.69 vs. 6.80%). In the case of the remaining OTUs, differences were recorded in the higher frequency in *O. alsatica* stigmas occurring in *Pantoea ananatis* (13.10 vs. 2.19%, *p* = 0.040), *Pseudomonas* spp. (9.36 vs. 5.43%) and Rickettsiales (10.89 vs. 1.68%, *p*  = 0.010). On the other hand, some OTUs reported a greater frequency in *O. bartlingii* stigmas represented by Enterobacteriaceae (13.51 vs. 1.64%) and *Pseudomonas fragi* (7.93 vs. 3.44%). In *O. bartlingii* stigma samples, these microorganisms were also noted as rare, which did not occur in *O. alsatica*, or occurred casually, such as *Paenibacillus* spp., *Sphingomonas* spp. (*p* = 0.030), and *Serratia marcescens* (Table 2).

The comparison of the composition of microorganisms in immature and mature stigmas of both species showed the existence of similarities and differences in the tested samples, supported by the conducted analyses (Table 2). At the phylum level, we observed a higher frequency of Actinobacteria in immature stigmas than in Proteobacteria, while in mature stigmas, the relationship was reversed (Fig. 1). The most abundant groups of *O. alsatica* stigmas were observed in both stages of flower development. However, in mature stigmas, the frequency of Enterobacteriaceae (from 0.25 to 3.04%) and *Pseudomonas fragi* (from 0.88 to 6.00%) increased as opposed to *Stenotrophomonas* spp. (from 9.06 to 4.31%), the frequency of which decreases by more than half. *Cellulosimicrobium* sp. occurred in both stages, but with the development of stigmas, their frequency decreased in *O. bartlingii* (78.23 vs. 25.64%) as well as *Stenotrophomonas* spp. (11.34 vs. 2.27%) and Rickettsiales (3.19 vs. 0.17%). However, there were numerous OTUs in mature stigmas that were not recorded in immature stigmas or are only casual and occasional, such as *Pantoea ananatis*, Enterobacteriaceae, *Pseudomonas* spp., *Pseudomonas fragi*, and *Paenibacillus* spp. (Table 2). In addition, in immature stigmas in both analysed species, the samples had a lower frequency of Enterobacteriaceae (0.28 vs. 14.88%, *p* = 0.029) and *Pseudomonas fragi* (0.99 vs. 10.38%, *p* = 0.029) than mature stigmas, but a higher frequency was recorded for *Cellulosimicrobium* sp. (68.25 vs. 35.62%, *p* = 0.013), and *Stenotrophomonas* spp. (10.20 vs. 3.29%, *p* = 0.001).

The fungal communities colonizing the stigmas of both species were more diverse than the bacterial profile (Table 3). In the case of interspecies differentiation, the high abundance similar frequency values were recorded for *Mycosphaerella tassiana* (14.05 vs. 15.64%), Pleosporales (7.24 vs. 8.04%), and *Chalastospora ellipsoidea* (3.83 vs. 5.31%). In *O. alsatica* stigmas, higher abundance of *Aureobasidium pullulans* (14.43 vs. 5.46%, *p* = 0.003), *Malassezia restricta* (11.46 vs. 5.07%, *p* = 0.011), *Volucrispora graminea* (6.29 vs. 0.98%, *p* = 0.018), and *Taphrina tormentillae* (3.16 vs. 0.22%, *p* = 0.014) were found than in *O. bartlingii*. In addition, *Tetracladium* spp. (40.82%) and Ceratobasidiaceae (10.34%) with high abundance were recorded only in *O. bartlingii* in a single sample. In relation to *O. alsatica*, higher abundance of *Cladosporium delicatulum* (7.71 vs. 17.44%, *p* = 0.005) and *Itersonilia pannonica* (0.04 vs. 2.58%, *p* = 0.027) were observed in *O. bartlingii* stigmas.

Analysing the fungal microbiome at different stages of development of the pistil stigmas in the case of *O. alsatica* OTUs, such as *Malassezia restricta*, reduced frequency was observed in relation to mature stigmas (20.18 vs. 2.74%). Interestingly, a large group of fungi occurred at a similar frequency at both stages of stigma development, i.e., *Mycosphaerella tassiana* (11.54 vs. 16.56%), *Cladosporium delicatulum* (7.90 vs. 7.51%), *Aureobasidium pullulans* (15.80 vs. 13.05%), and *Vishniacozyma victoriae* (2.60 vs. 3.25%). The frequency of *Chalastospora ellipsoidea* in mature stigmas increased (from 1.54 to 6.13%) as did Pleosporales (from 4.51 to 9.97%) in contrast to the abundance of *Volucrispora graminea* (from 7.92 to 4.67%) (Table 3). On the other hand, the fungal microbiome of *O. bartlingii*, similar to the bacterial one, but is more various in the presented stages of development of stigmas. However, similar values of these microorganisms for both stages were found, i.e., *Mycosphaerella tassiana* (13.82 vs. 17.46%), *Cladosporium delicatulum* (15.44 vs. 19.44%), and *Aureobasidium pullulans* (5.42 vs. 5.51%). For immature stigmas of *O. bartlingii*, the abundance of *Malassezia restricta* was higher (8.88 vs. 1.26%), as were Pleosporales (11.53 vs. 4.55%) and *Chalastospora ellipsoidea* (8.12 vs. 2.50%). In addition, OTUs such as *Tetracladium* spp. were found only in mature stigmas in a single sample (40.82%), as opposed to Ceratobasidiaceae, which was recorded at a high frequency (10.34%) only in immature stigmas (Table 3). In immature stigmas in both analysed species, the samples had a higher frequency of *Malassezia restricta* (14.53 vs. 2.00%, *p*  = 0.001) than mature stigmas, and most recorded fungi had a similar frequency in both stages.

**Figure 2:**
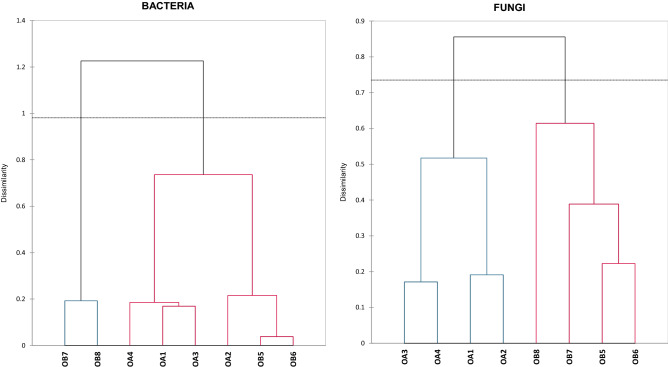
Agglomerative Hierarchy Clustering analysis (Ward’s method based on Bray‒Curtis dissimilarity matrix) of distance between phyla and order-representing microbiomes inhabiting *Orobanche alsatica* (OA) and *O. bartlingii* (OB) in immature stigmas from closed flowers (1, 2, 5, 6) and mature stigmas from opened flowers (3, 4, 7, 8) (fungi on the right, bacteria on the left).

According to the results of the agglomeration hierarchical grouping analysis, a clear grouping of stigma samples into two clades for bacterial and fungal microorganisms was observed (Fig. 2). By analysing the arrangement of the AHC dendrogram for the distribution of variants, it was observed that two groups were formed in the clade consisting of bacteria. The first one consists of two subgroups that correspond to immature and mature stigmas of *O. alsatica* (samples OA1, OA3 and OA4) as well as the other which consists also of two subgroups with two variants corresponding to *O. bartlingii* immature stigmas (OB5, OB6) and a variant of *O. alsatica* immature stigmas (OA2). The second clade is represented by *O. bartlingii* variants of mature stigmas (OB7, OB8). In the case of a dendrogram showing fungal microorganisms, the first clade consisted of two groups, the first consisting of *O. alsatica* immature stigmas (OA1, OA2) and mature stigmas (OA3 and OA4). The second clade consisted of a group with a proportionally increasing dissimilarity consisting of *O. bartlingii* immature stigmas (OB5, OB6) to *O. bartlingii* mature stigmas (OB7, OB8).

**Figure 3:**
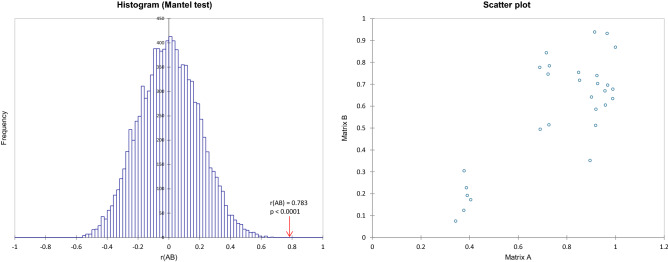
Mantel test for comparison of both correlation matrices (bacteria vs. fungi) using the Monte Carlo method.

To determine the correlations between bacterial and fungal microorganisms colonizing stigmas, the Mantel test (linear correlation for Pearson) was performed (Fig. 3). A correlation coefficient of r = 0.783 was obtained at α = 0.05. However, the calculated value of *p* < 0.0001 was lower than the significance level, which indicates the presence of correlations between the matrices under investigation.

**Figure 4:**
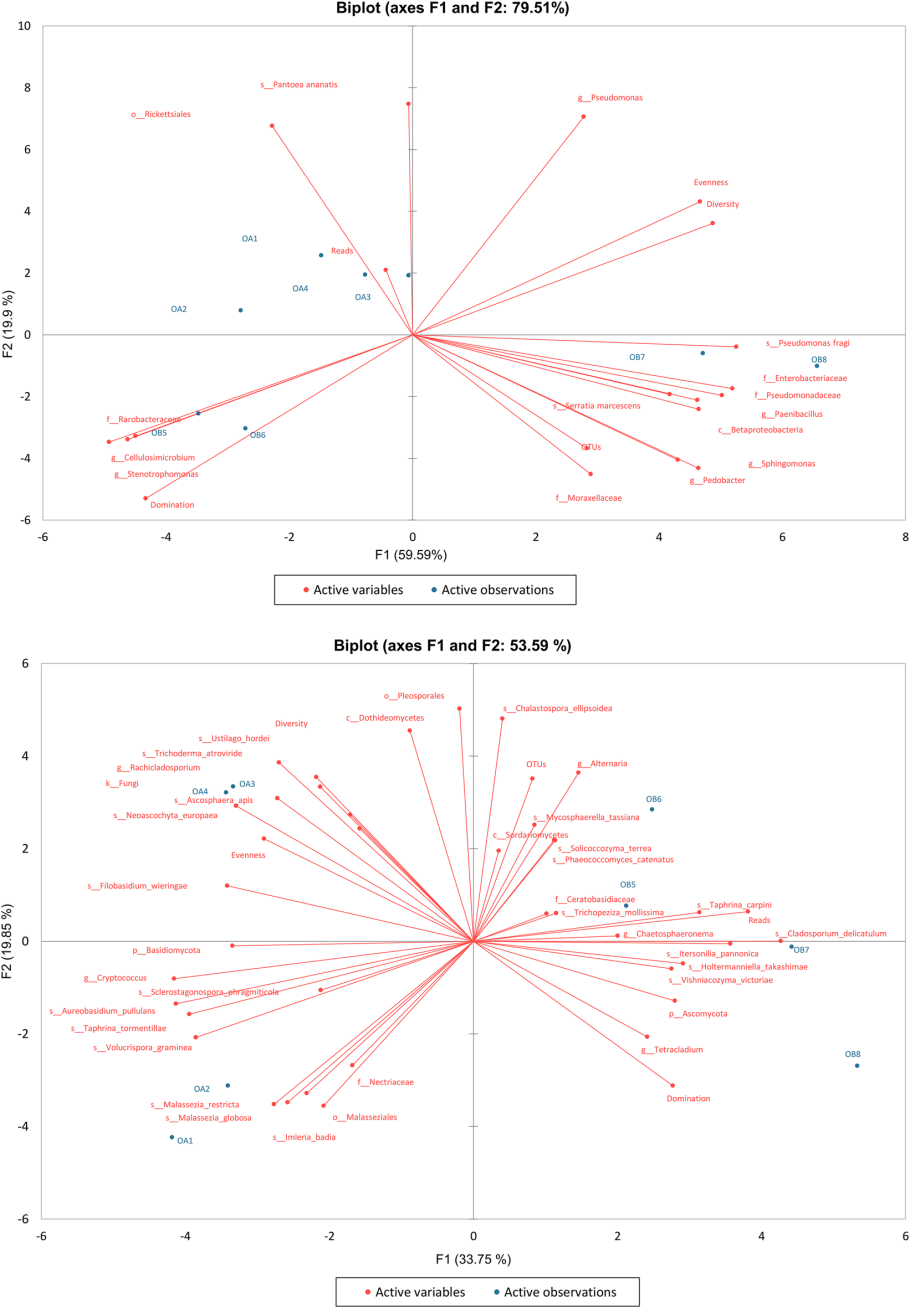
Relation between the most abundant orders of bacteria (top) and fungi (bottom) and their influence on samples of *Orobanche alsatica* (OA) and *O. bartlingii* (OB) in immature stigmas from closed flowers (1, 2, 5, 6) and mature stigmas from opened flowers (3, 4, 7, 8), according to PCA.

Additionally, the biplot PCA results made it possible to ungroup the composition of microorganisms between samples of *O. alsatica* and *O. bartlingii* immature and mature stigmas. The biplot PCA for bacteria and fungi is shown separately in Fig. 4. In each case, the first two factors (F1 and F2) allow us to represent high values of the initial variability of the data, i.e., 79.51% for bacteria and 53.59% for fungi. The calculated PCA indicates almost total separation of the investigated samples of stigma containing bacterial microbiomes. The variants of *O. alsatica* stigmas (OA1–OA4) were relatively similar to each other and correlated with *Pantoea ananatis* and Rickettsiales. For samples of *O. bartlingii* immature stigmas (OB5, OB6), there were correlations between Rarobacteraceae, *Cellulosimicrobium* and *Stenotrophomonas* genera. The samples of *O. bartlingii* mature stigmas (OB7, OB8) correlated with *Pseudomonas fragi*, Enterobacteriaceae, Pseudomonadaceae, *Paenibacillus* spp., Betaproteobacteria and *Serratia marcescens*. Detailed biplot PCA results for fungi showed correlations with Nectriaceae, Malasseziales, *Imleria badia*, *Malassezia restricta*, and *M. globosa* with *O. alsatica* immature stigma samples (OA1, OA2) (Fig. 4). For *O. alsatica*, mature stigma samples (OA3, OA4) were correlated with *Neoascochyta europaea*, *Ascosphaera apis*, Fungi, *Rachicladosporium* sp., *Trichoderma atroviride*, and *Ustilago hordei*. It was also observed that *Alternaria* spp., *Mycosphaerella tassiana*, Sordariomycetes, *Solicoccozyma terrea*, *Phaeococcomyces catenatus*, Ceratobasidiaceae, *Trichopeziza mollissima*, *Taphrina carpini* and *Chaetosphaeronema* sp. were correlated with each other in *O. bartlingii* immature stigma samples (OB5, OB6). The location of *O. bartlingii* mature stigma samples (OB7, OB8) was mainly influenced by *Cladosporium delicatulum*, *Itersonilia pannonica*, *Holtermanniella takashimae*, *Vishniacozyma victoriae*, Ascomycota and *Tetracladium* spp.

In relation to fungi, the PCA showed the separation of *O. alsatica* samples into those belonging to immature stigmas (OA1, OA2) and mature stigmas (OA3, OA4), which suggest a greater diversity of these microorganisms than bacteria. The results of the analysis of the fungal community indicates total separation of *O. alsatica* stigma samples from *O. bartlingii* samples (OB5–OB8). Additionally, some of the pathogenic bacteria or fungi observed in stigma samples may be suppressed by microorganisms able to biocontrol. Based on the PCA, it was possible to establish an overall differentiation between the samples taking into account all observations and the relationships between these variables included in these studies. Bacteria of all *O. alsatica* samples were similar and dominated by *Pantoea ananatis* and Rickettsiales. On the other hand, the samples from immature and mature *O. bartlingii* stigmas were clearly different from each other and from the samples of *O. alsatica*. *O. bartlingii* samples of immature stigmas were characterized by the domination of three groups of potentially symbiotic microorganisms and were negatively correlated with the diversity and evenness indices. It was also observed that in the case of mature stigmas samples of *O. alsatica*, a potentially antagonistic fungi *Trichoderma artoviride* was present, and for these samples, no more Nectriaceae was observed, and the vector for *Ustilago hordeii* was also reduced. Nevertheless, *Rachicladosporium* sp. for which only *Alternaria* spp. was characteristic (long vector) in *O. bartlingii* immature stigma samples. On the other hand, *O. bartlingii* samples of mature stigmas had a mycobiome consisting of nonphytopathogenic fungi (saprotrophs or symbionts) or fungi of unknown function. When comparing the PCA microbiome of bacteria and fungi, it was observed that *O. alsatica* stigma samples had more characteristic fungal OTUs and only two bacterial OTUs, indicating the displacement of bacteria by fungi or the lack of a symbiotic nature of this plant species. On the other hand, the reluctant colonization of *O. alsatica* stigma samples by bacteria may open a niche for phytopathogens, as evidenced by *Malassezia* spp. (putative *Ustilago* spp*.*) and Nectriaceae (most representatives of this family are plant pathogens). The samples of mature stigmas of *O. bartlingii*, no typical fungal plant pathogens were found. Nevertheless, the presence of common plant growth-promoting and antagonistic bacteria has been confirmed: *Pseudomonas* spp., *Paenibacillus* spp., and *Sphingomonas* spp. A large proportion of bacteria (spores, Enterobacteriaceae, *Pantoea* spp., *Erwinia* and *Serratia* spp. and *Pseudomonas* spp.), and fungi (*Alternaria* spp. and *Fusarium* spp.) are typical plant endophytes and have also been identified in this paper. The high diversity of potentially endophytic and migratory species (mainly *Ustilago* spp. and *Cladosporium* group) probably proves (excluding *Alternaria* spp.) the differences in the susceptibility of samples of both species to infection by phytopathogens are also evidenced by the coexistence of these plants with likely antagonistic endophytes. The results of PCA indicate an inverse relation between the presence of *Alternaria* spp. relative to *Cellulosimicrobium* sp. and *Stenotrophomonas* spp. in immature stigma samples of *O. bartlingii* (OB5, OB6) as well as Pseudomonadaceae, *Pseudomonas fragi* and *Paenibacillus* spp. in mature stigma samples (OB7, OB8).

## Discussion

The distances between the first and second principal components identified by PCA as well as AHC analysis (Figs. 3, 4) allow us to state that stigma samples are unique in terms of the composition of recognized microorganisms. This implies that dynamic microbial communities are different between *Orobanche alsatica* and *O. bartlingii* and underwent significant changes during flower development. This suggests that stigmas are a place of dynamic dependencies and competition between beneficial and pathogenic microorganisms. Analysis of the structure of the microbiome of bacteria and fungi in *O. alsatica* and *O. bartlingii* stigmas allowed changes in the composition of the community to be detected between the various stages of flower development. The use of indices such as Simpson’s dominance and Shannon diversity indices confirm the assumption of greater variation within mature stigmas. The most diverse in terms of the frequency of microorganisms were samples of mature stigmas of *O. bartlingii* (OB7, OB8). The fungal communities were more diverse than the bacterial communities in both species and in both stages of development (Table 1). In the case of the bacterial profile, there were several OTUs that were classified as eudominant, dominant or subdominant, and the rest were much less frequent. On the other hand, fungal microorganisms were more diverse in the case of OTUs being subdominant or rare. However, the similarity of the occurrence and general frequency of especially eudominants and dominants of bacteria and fungi (e.g., *Cellulosimicrobium* sp., *Mycosphaerella tassiana*, *Cladosporium delicatulum*) between *O. alsatica* and *O. bartlingii* is extremely interesting. It should be emphasized that the populations of both species are separated by approximately 90 km.

Interestingly, substantial abundance of Actinobacteria, Proteobacteria (e.g., genera *Pantoea*, *Pseudomonas*, *Stenotrophomonas* and Moraxellaceae) and Firmicutes (*Paenibacillus* spp.) in the stigmas of apple trees^27–31^ were also confirmed to have been detected in the stigmas of *Orobanche* tested (Fig. 1). The temporal dynamics in apple stigma microbiome revealed a diverse bacterial community evolving into a community dominated by two families within the phylum Proteobacteria, the Pseudomonadaceae and Enterobacteriaceae^28^, which was also confirmed in our results in mature stigmas. *Pantoea agglomerans* strain E325 also had biocontrol activity against *Erwinia amylovora* on apple flower stigmas^39^. In addition, petals of *Saponaria officinalis* L. and *Lotus corniculatus* L. were dominated by members of the family Enterobacteriaceae (higher frequency of *Serratia* sp.)^40^. *Serratia marcescens* was also identified in mature stigmas of *O. bartlingii*. We observed also that the phyla and OTUs identified similar to soil microbiomes, especially genera *Pseudomonas*, *Paenibacillus*. These and other taxa (genera *Bacillus*, *Methylobacterium*, *Rhizobium*) were also identified in papers about microbiome flowers^24^. The higher prevalence of this group of microorganisms may be due to the fact that parasitic plants have a specific life cycle in which they spend most of their time in the rhizosphere as seeds which remain viable in the soil for many years. The communities of fungi, especially yeasts found in nectar, contain similar groups identified in stigmas in particular, e.g., *Aureobasidium pullulans*, *Cryptococcus* spp., *Filobasidium wieringae*, and *Vishniacozyma victoriae*. This may be due to the fact that stigmas, like nectaries, are habitats extremely rich in various types of nutrient substances^15,16^. *Cellulosimicrobium* sp. which was a eudominant or dominant in study stigmas was also found in floral nectar^41^. Some plant pathogens are able to inhibit the development of stigmas and thus, more dangerously, prevent the correct pollination process (e.g., *Salmacisia buchloëana*). The pistil smut fungus shifts sex ratios to be nearly 100% phenotypically hermaphroditic^28,42,43^. However, this study did not confirm the presence of these pathogens colonizing stigma. Before the flower opens, the sesquiterpene products are emitted in the bud headspace and are then absorbed and accumulated by the stigma and anthers, which supports the proper development of these structures of petunia and provides protection against microorganisms^44^. Additionally, the stigma of the pistil provides a specific microhabitat and may be an attractive source of nutrients, supporting the growth of the population of microorganisms, which may also affect the pollination process itself, stimulating the production of specific metabolites and phytohormones as well as affecting pollinators. Moreover, an extremely interesting issue is to provide information on which insects support the colonization of bacteria and fungi on stigmas. As other studies show, not only typical pollinators can be responsible for this but also the fauna that inhabit flowers on a daily basis^45^.

Orobancheae species are poorly known in terms of the bacterial and fungal microbiota that inhabit them^32–38^. These microorganisms can have the potential to mitigate the impacts of unfavourable environmental conditions, as well as the negative effects of plant pathogen infections^19,35,36^. Due to the dynamic and brief development of species from the Orobanchaceae family, microorganisms can respond to and potentially aid the current needs of these heterotrophic plants; for example, microorganisms in the sunflower rhizosphere affect parasitic seed germination and growth^46^ as well as endophytic bacteria in seeds of *Cistanche armena* are able to improve the tolerance of parasitic plants under stress conditions in their natural habitat^37^. On the other hand, for the few parasitic plants that are dangerous weeds of economic importance, microbial communities may play an important role in mitigating the negative effects of infections caused by these plants. Thus, the production of various metabolites by microorganisms can support their host plant in different ways^32,47^. Notably, interactions among these species are significant determinants of the overall composition and function of plant microbiota^34^.There is no data on microorganisms colonizing the stigma of the pistil, which may be of key importance in their adaptation to the environment and reproductive biology, especially in the pollination phase. The development of the stigmas of the studied species of holoparasitic plants takes only a few days. In this short period of time, crucial for the plant, a number of mechanisms are generated that help them communicate with the environment in response to specific stimuli. In addition, a variety of substances produced within the stigmas are important in relationships with other organisms that use the same habitat island. The possibility of colonization of microorganisms from the external environment in mature stigmas occurs both day and night because the flowers of the tested Orobanchaceae species remain constantly open. This abundant and specialized ecological community of the stigma consists of commensal as well as symbiotic and pathogenic microorganisms. These microorganisms can be transferred horizontally through environmental transfer via pollinator, atmospheric or soil contamination and vertically during the parasitic plant life cycle^48^. Seed-fungal communities have been observed to be transmitted horizontally by the environment and soil versus seed-bacterial communities, which had mostly vertical transmission^49^⁠. However, insect pollination is an ecological process involved in the transmission of bacteria from flowers to seeds; thus, the seed microbiota consists of microorganisms inhabiting not only the plant vascular tissues but also the flowers^50^. As microorganisms can interact with parasitic plants, notably during the early stages, they may have played a role in specialization. This connection allows for the exchange of various substances and microorganisms that inhabit the internal tissues of plants. There is a mutual transfer of microorganisms and homogenization between the host and the parasite during the interaction^32,34^. The presence of rhizosphere-related bacteria in mature stigmas of *O. bartlingii* may also be associated with soil contamination or/and the presence of a parasitic plant in the anthropogenic area. This is because the seeds of parasitic plants are disseminated in the soil where they can lie for decades. This can increase competition between soil microbiota and seed-born pioneer endophytes and modify the microbial profile^35^. Enterobacteriaceae species, detected in seeds, suggest a possible bacterial transmission to the seed through insect pollinators^51^, also recorded in our study in mature stigmas at a higher frequency. In mature stigmas, horizontal transmission could be due to environmental microbial deposition on flowers and pollinators. It is noteworthy that using bumblebees as vectors of various biocontrol agents is becoming increasingly popular. The potential of the yeast-like biocontrol fungus *Aureobasidium pullulans* vectored by bumblebees (*Bombus terrestris*) has been investigated, which significantly reduced the fungal pathogens. The performance and activity of the bees were not negatively affected by *A. pullulans*^52^.

## Conclusions

Holoparasitic plants are a specific group of plants that are obligately host-dependent. In this case, there is also a likelihood that these plants benefit from the presence of bacteria and fungi colonizing the stigmas that cooperate or compete with each other during the development of stigmas, lasting only several days. The variability of microorganisms between immature stigmas from closed flowers and mature stigmas from opened flowers shows that thus far unexplored niches are dynamic, so the whole plant is also able to respond to situations from the external environment. The presence of a greater variety of fungi may be related to the fact that the stigmas provide a convenient microhabitat and an attractive source of nutrients that are more suitable for the growth of fungi, including the symbiotic yeasts that dominate the fungal profile of the stigmas. Thus, the stigmas of parasitic plants, although small, constitute a very rich microenvironment that is extremely diverse in terms of the presence of the described microorganisms. Furthermore, understanding the diversity and role of the stigma microbiome related to their reproductive biology can help to better protect these endangered species. To define the potential function and role of these bacterial and fungal microbiomes for parasitic plants, especially for the development of stigma and pollination, more research is needed.

## Materials and methods

### Study species

**Figure 5:**
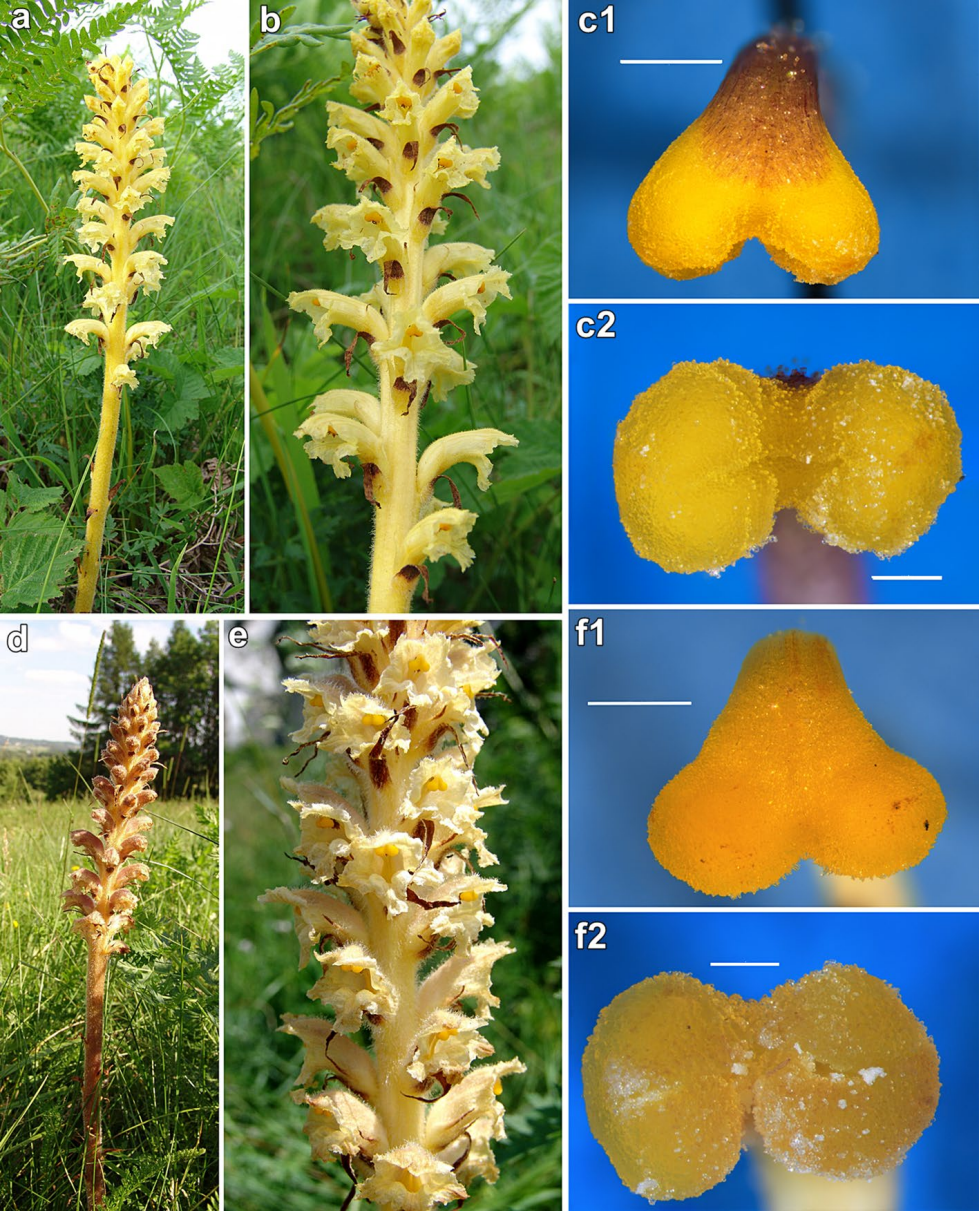
Studied holoparasitic *Orobanche alsatica* (**a**, **b**, **c**) and *O. bartlingii* (**d**, **e**, **f**): (**a**, **d**) general habit; (**b**, **e**) closed and opened flowers, phot. R. Piwowarczyk; (**c**, **f**) ZOOM micrographs of two-lobed mature stigmas of *O. alsatica* and *O. bartlingii* with numerous papillae covered with a viscous secretion. Scale bars: (**c1**, **f1**) 1000 µm; (**c2**, **f2**) 500 µm. Phot. K. Ruraż.

*Orobanche alsatica* and *O. bartlingii* mostly prefer xerothermic grasslands and thermophilous fringe communities, woodland glades, bordering open forests, also occurring in wasteland and abandoned fields, often on alkaline, clay or loess soils^6,53,54^. *Peucedanum cervaria* (L.) Lapeyr. is the host plant of *O. alsatica*, while *O. bartlingii* parasitizes *Libanotis pyrenaica* (L.) Bourg. in its localities in Poland. These parasitic plants form scaly simple stems with a single nonbranched inflorescence, which takes up one-quarter to half of the emerged stems. The flowers are bisexual, zygomorphic, and insect pollinated. The species flowers in June and the first half of July. The stigma of these species consists of two lobes and is yellow^3,6,55^ (Fig. 5). Significant differences between both species in morphology^3,6,53^, seed and pollen micromorphology^56–58^, as well as molecular differences^59^, have been presented as well.

### Plant material and sample collection

Field studies were carried out during the flowering period of *Orobanche alsatica* and *O. bartlingii* in Poland in June 2021. Samples of *O. alsatica* stigma were collected from Grabina Mt. near Kielce (Małopolska Upland) and samples of *O. bartlingii* in Podzamcze near Ogrodzieniec, in the vicinity of the Ogrodzieniec Castle (Kraków-Częstochowa Upland). The localities are approximately 90 km apart. The distribution of both species in Poland and their habitats, communities, hosts, and taxonomic problems were discussed^53,60^. For research, we chose parasitic plant populations from the localities mentioned above, among the most numerous in Poland. *O. alsatica* and *O. bartlingii* are endangered and vulnerable species and partially protected in Poland, and only a limited number of individuals (10) of each species have been authorized to be sampled for this project.

The flowers of both species were placed into sterile plastic tubes. Samples of parasitic plants with fully opened and closed petal flowers (flower bud) were selected separately. The samples were transported the same day to the laboratory and stored at 4 ± 0.5 °C until the analysis, which was carried out within 24 h. Approximately 250 stigmas from fully opened (mature stigmas) and closed petal flowers (immature stigmas) (approximately 1 g of each sample) were immediately dissected, with sterile measures, from the flowers into plastic tubes and stored at − 80 ± 0.5 °C until microbial analysis. A total of eight samples were used for further analyses, four per species, i.e., two for closed (buds) petal flowers (immature stigmas) and two for opened flowers (mature stigmas). For these samples, the pistil was cut as close as possible to the base of the stigma. The plant materials were identified by Renata Piwowarczyk and collected by Renata Piwowarczyk and Karolina Ruraż. Sampling of plant material was prepared with permission no. WPN.I.6400.3.1.2021.AD (*O. alsatica*) and WPN.6400.4.2021.MS1.1 (*O. bartlingii*) from the Regional Directors for Environmental Protection in Poland. Experimental research and field studies on plants, including the collection of plant material was complied with relevant institutional, national, and international guidelines and legislation, and necessary permissions were obtained. Voucher material was deposited in the Herbarium of Jan Kochanowski University in Kielce (KTC, acronym according to Thiers^61^). The plant names were updated based on The International Plant Names Index (IPNI)^62^.

### Sequencing

Laboratory analyses (DNA extraction and PCR amplification) were performed by A&A Biotechnology (Poland), whereas NGS library preparation and Illumina and Sanger sequencing (the 16S rRNA and ITS products) were conducted by Macrogen (The Netherlands). The microbial communities colonizing the analysed samples were examined by sequencing the V3-V4 region of the 16S rRNA gene and ITS region. The gene fragments were amplified with the PCR primers recommended for the Illumina technique. Primers ITS3F (GCATCGATGAAGAACGCAGC) and ITS4R (TCCTCCGCTTATTGATATGC) for fungal ITS library, 341F (CCTACGGGNGGCWGCAG) and 805R (GACTACHVGGGTATCTAATCC) for bacterial 16S rRNA libraries were employed. The primers were developed by adding Illumina adapter overhang nucleotide sequences to the PCR primers used for this sequencing platform. Amplicons were indexed using the Nextera^®^ XT Index Kit according to the manufacturer's instructions. DNA was sequenced in Illumina MiSeq in 2 × 250 paired-end mode. Sequencing results were saved in FASTQ files and uploaded to the MetaGenome Rapid Annotation Subsystems Technology (MG-RAST) server for analysis^63^. Each file underwent quality control (QC), which included quality filtering (removing sequences with ≥ 5 ambiguous base pairs) and length filtering (removing sequences with a length ≥ 2 standard deviations from the mean). Sequences below three reads (singletons) or with abundance less than 0.0005% were removed after generating the ASV table. Raw data Stats of the Q20/Q30 values and data yield was presented in Supplementary Table 1. Illumina metagenomic datasets are available at MG-RAST under accession numbers 4,978,360.3 to 4,978,367.3 for bacteria and 4,978,430.3 to 4,978,444.3 for fungi. The results were obtained using QIIME 2^64^ and Pipeline based on the SILVA^65^ or UNITE^66^ databases.

### Statistical calculations

The OTU compositions of bacteria and fungi were analysed. The taxonomic diversities of the analysed OTUs were determined with the use of Simpson dominance (λ), Shannon diversity index (H’), and Pielou’s evenness index (J′)^67–69^. Domination classes were determined according to previous work Przemieniecki et al.^70^ for bacteria and Kurowski et al.^71^ for fungi.

The normality of the distribution of the obtained results was tested (Shapiro–Wilk test) and equality of variances was tested Levene’s Test. Then, t-test or the Mann–Whitney U test for normally or non-normally distributed datasets was used to evaluate the statistical differences of microorganisms colonizing *Orobanche alsatica* versus *O. bartlingii*, as well as immature stigmas from closed flowers versus mature stigmas from opened flowers.

Global relationships were calculated using principal component analysis (PCA) and agglomerative hierarchical clustering (AHC). Correlation matrices for principal component analysis were estimated using Pearson's correlation coefficient. AHC dissimilarities were calculated based on the Bray‒Curtis method, and dendrograms were constructed based on Ward’s method. The Mantel test for comparison of both correlation matrices (bacteria vs. fungi) was performed using the Monte Carlo method (10,000 permutations) for *P* values prediction. Statistical calculations were made with XLSTAT program^72^.

## Supplementary Information

Supplementary Information.

## Data Availability

The raw Illumina sequencing data generated in this study are available in the MG-RAST database (accession number: from 4,978,360.3 to 4,978,367.3 for bacteria and from 4,978,430.3 to 4,978,444.3 for fungi, https://www.mg-rast.org/linkin.cgi?project=mgp104278; https://www.mg-rast.org/linkin.cgi?project=mgp104297).
