# Supplementary material for: Interspecies and temporal dynamics of bacterial and fungal microbiomes of pistil stigmas in flowers in holoparasitic plants of the Orobanche series Alsaticae (Orobanchaceae)
Source: Sci Rep. 2023 Apr 25;13:6749. doi: 10.1038/s41598-023-33676-0 (PMC10130099; doi:10.1038/s41598-023-33676-0)
Supplement: Supplementary file 1 — Supplementary Information. [file 41598_2023_33676_MOESM1_ESM.docx]

**Interspecies and temporal dynamics of bacterial and fungal** **microbiomes of pistil stigmas in flowers in holoparasitic plants of the *Orobanche* series *Alsaticae* (Orobanchaceae)**

**Karolina Ruraż^a,*^, Sebastian Wojciech Przemieniecki^b^, Renata Piwowarczyk^a^**

^a^*Center for Research and Conservation of Biodiversity, Department of Environmental Biology, Institute of Biology, Jan Kochanowski University, Uniwersytecka 7, PL-25-406 Kielce, Poland; karolina.ruraz@ujk.edu.pl, ORCID: 0000-0003-4831-7712; piwowarczyk@ujk.edu.pl, ORCID: 0000-0003-0507-7835*

^b^*Department of Entomology, Phytopathology and Molecular Diagnostics, University of Warmia and Mazury in Olsztyn, Prawocheńskiego 17, PL-10-720 Olsztyn, Poland;* [*sebastian.przemieniecki@uwm.edu.pl*](mailto:sebastian.przemieniecki@uwm.edu.pl)*, ORCID: 0000-0003-1222-0310*

*Corresponding author

Center for Research and Conservation of Biodiversity, Department of Environmental Biology, Institute of Biology, Jan Kochanowski University, Uniwersytecka 7, PL-25-406 Kielce, Poland; e-mail address: karolina.ruraz@ujk.edu.pl

Supplementary Tab. S1

Table S1. Raw data Stats of 16 samples from *Orobanche alsatica* (OA) and *O. bartlingii* (OB) in immature stigmas from closed flowers (1, 2, 5, 6) and mature stigmas from opened flowers (3, 4, 7, 8)

| **Sample ID** | **Total read bases (bp)*** | **Total reads*** | **GC(%)*** | **AT(%)*** | **Q20(%)*** | **Q30(%)*** |
| --- | --- | --- | --- | --- | --- | --- |
| gOA1_16S | 57,854,608 | 192,208 | 55.87 | 44.13 | 92.19 | 84.19 |
| gOA1_ITS | 63,253,946 | 210,146 | 49.46 | 50.54 | 92.0 | 84.4 |
| gOA2_16S | 48,978,720 | 162,720 | 56.35 | 43.65 | 92.0 | 83.98 |
| gOA2_ITS | 77,211,918 | 256,518 | 49.98 | 50.02 | 92.64 | 85.1 |
| gOA3_16S | 54,219,130 | 180,130 | 55.76 | 44.24 | 91.92 | 83.76 |
| gOA3_ITS | 67,765,334 | 225,134 | 48.29 | 51.71 | 91.79 | 84.11 |
| gOA4_16S | 50,341,046 | 167,246 | 55.77 | 44.23 | 91.41 | 83.02 |
| gOA4_ITS | 72,637,922 | 241,322 | 47.48 | 52.52 | 92.58 | 85.79 |
| gOB5_16S | 55,926,402 | 185,802 | 56.45 | 43.55 | 90.72 | 81.95 |
| gOB5_ITS | 70,182,364 | 233,164 | 48.23 | 51.77 | 92.91 | 85.62 |
| gOB6_16S | 51,937,550 | 172,550 | 56.56 | 43.44 | 91.81 | 83.66 |
| gOB6_ITS | 77,656,194 | 257,994 | 47.5 | 52.5 | 93.7 | 86.96 |
| gOB7_16S | 48,105,820 | 159,820 | 54.88 | 45.12 | 91.79 | 83.61 |
| gOB7_ITS | 77,484,022 | 257,422 | 47.65 | 52.35 | 92.9 | 85.87 |
| gOB8_16S | 53,900,672 | 179,072 | 54.84 | 45.16 | 92.25 | 84.37 |
| gOB8_ITS | 75,982,634 | 252,434 | 47.41 | 52.59 | 93.74 | 87.03 |

***Total read bases**: Total number of bases sequenced. **Total reads**: Total number of reads. For Illumina paired-end sequencing, this value refers to the sum of read 1 and read 2. **GC(%)**: GC content. **AT(%)**: AT content. **Q20(%)**: Ratio of bases that have phred quality score of over 20. **Q30(%)**: Ratio of bases that have phred quality score of over 30.
